# Investigating a Potential Causal Relationship Between Maternal Blood Pressure During Pregnancy and Future Offspring Cardiometabolic Health

**DOI:** 10.1161/HYPERTENSIONAHA.121.17701

**Published:** 2021-11-17

**Authors:** Geng Wang, Laxmi Bhatta, Gunn-Helen Moen, Liang-Dar Hwang, John P. Kemp, Tom A. Bond, Bjørn Olav Åsvold, Ben Brumpton, David M. Evans, Nicole M. Warrington

**Affiliations:** The University of Queensland Diamantina Institute (G.W., G.-H.M., L.-D.H., J.P.K., T.A.B., D.M.E., N.M.W.), The University of Queensland, Brisbane, Australia.; Institute of Molecular Bioscience (L.-D.H., J.P.K., D.M.E., N.M.W.), The University of Queensland, Brisbane, Australia.; K.G. Jebsen Center for Genetic Epidemiology, Department of Public Health and Nursing, NTNU, Norwegian University of Science and Technology, Trondheim, Norway (L.B., G.-H.M., B.O.A., B.B., N.M.W.).; Institute of Clinical Medicine, Faculty of Medicine, University of Oslo, Norway (G.-H.M.).; Population Health Sciences, Bristol Medical School (G.-H.M., T.A.B.), University of Bristol, United Kingdom.; Medical Research Council Integrative Epidemiology Unit (J.P.K., T.A.B., D.M.E., N.M.W.), University of Bristol, United Kingdom.; Department of Endocrinology, Clinic of Medicine (B.O.A.), St Olavs Hospital, Trondheim University Hospital, Norway.; Clinic of Medicine (B.B.), St Olavs Hospital, Trondheim University Hospital, Norway.; HUNT Research Center, Department of Public Health and Nursing, NTNU, Norwegian University of Science and Technology, Levanger, Norway (B.O.A., B.B.).

**Keywords:** adult children, blood pressure, cardiometabolic risk factors, cohort studies, genotype, pregnancy, Mendelian randomization analysis

## Abstract

Observational epidemiological studies have reported that higher maternal blood pressure (BP) during pregnancy is associated with increased future risk of offspring cardiometabolic disease. However, it is unclear whether this association represents a causal relationship through intrauterine mechanisms. We used a Mendelian randomization (MR) framework to examine the relationship between unweighted maternal genetic scores for systolic BP and diastolic BP and a range of cardiometabolic risk factors in the offspring of up to 29 708 genotyped mother-offspring pairs from the UKB study (UK Biobank) and the HUNT study (Trøndelag Health). We conducted similar analyses in up to 21 423 father-offspring pairs from the same cohorts. We confirmed that the BP-associated genetic variants from the general population sample also had similar effects on maternal BP during pregnancy in independent cohorts. We did not detect any association between maternal (or paternal) unweighted genetic scores and cardiometabolic offspring outcomes in the meta-analysis of UKB and HUNT after adjusting for offspring genotypes at the same loci. We find little evidence to support the notion that maternal BP is a major causal risk factor for adverse offspring cardiometabolic outcomes in later life.

## Abstract 2

Supplemental Digital Content is available in the text.

Observational epidemiological studies using multivariable regression have shown that gestational hypertensive disorders are associated with increased risk of offspring cardiometabolic diseases in later life, including cardiovascular diseases and type 2 diabetes.^1–5^ These associations could be due to intrauterine effects (ie, developmental programming), in which case intervening to prevent gestational hypertensive disorders could also lower cardiometabolic risk in the offspring.^6^ However, although maternal blood pressure (BP) during pregnancy is associated with offspring cardiometabolic risk factors, in particular offspring BP,^7^ sibling studies have indicated that the associations could be explained by confounding due to postnatal environmental factors or inherited genetic variants instead of intrauterine programming.^8–10^ Consequently, definitive evidence as to whether increased maternal BP during pregnancy has long-term impacts on offspring cardiometabolic health in human populations is lacking. Understanding this relationship will help determine whether intervening on maternal BP during pregnancy will combat the rising incidence of offspring cardiometabolic diseases in adulthood.

Mendelian randomization (MR) is an epidemiological method used to estimate the causal relationship between a modifiable environmental exposure of interest and a medically relevant trait or disease.^11,12^ Mendel’s Laws of Inheritance (ie, segregation, independent assortment) mean that genetic variants are often less susceptible to confounding and reverse causality than the variables used in traditional observational epidemiological studies.^13^ We have previously developed a MR framework to investigate the potential maternal exposures on offspring’s health and disease in later life^14^ (Figure S1A).

Most previous MR studies investigating the relationship between early life environmental exposures and later-life cardiometabolic traits and diseases have not distinguished between maternal and offspring genetic effects, which has complicated interpretation of the results of such investigations.^15–18^ This has partly been due to the paucity of cohorts world-wide with genotyped mother-offspring pairs and offspring of advanced age, hindering the estimation of maternal genetic effects on offspring who have developed cardiometabolic disease. In the current study, we addressed these issues by performing a genetic association study in up to 29 708 genotyped mother-offspring pairs and up to 21 423 father-offspring pairs from the UKB study (UK Biobank)^19^ and HUNT study (Trøndelag Health).^20^ Specifically, we regressed offspring cardiometabolic risk factors on maternal genetic risk scores (GRSs) for BP while simultaneously conditioning on offspring genotypes at the same loci, thereby accounting for the potential contaminating influences of genetic pleiotropy through the offspring genome.^14^ Associations between maternal GRSs and offspring outcomes would be consistent with a causal effect of maternal BP on the offspring outcomes.

## Methods

### Data Availability

Human genotype and phenotype data from the UKB on which the results of this study were based were accessed with accession ID 12703 and 53641. The genotype and phenotype data are available upon application to the UKB (http://www.ukbiobank.ac.uk/). Phenotype and genotype data from the ALSPAC (Avon Longitudinal Study of Parents and Children) and HUNT studies are archived centrally with the corresponding cohort studies. Individual-level data can be made available to researchers upon application to the resources. Requirements for data access to the UKB, ALSPAC,^21–23^ and the HUNT studies are described at https://www.ukbiobank.ac.uk/, http://www.bristol.ac.uk/alspac/, and www.ntnu.edu/hunt/, respectively.

### UKB Study

The UKB study is a study of over 500 000 volunteers (with 5.45% response rate of those invited^24^), recruited from across the United Kingdom at age 40 to 69 years between 2006 and 2010, with a broad range of health-related information and genome-wide genetic data^25^ (further details are provided in the Supplemental Material^26,27^). Only individuals of European ancestry were included in the present study (Supplemental Material).

Parent-offspring relationships were inferred by the KING software using genotyping data^28^ (Supplemental Material). After cleaning, there were 4119 mother-offspring pairs and 1829 father-offspring pairs available for analysis (not all offspring had phenotypic data available for each of the cardiometabolic risk factors of interest, so the numbers are smaller for each specific analysis; Table S1).

### The HUNT Study

The HUNT is a large population-based study of ≈240 000 participants (with >50% response rate of those invited) with a broad range of health-related information and genome-wide genetic data^20,29^ (Supplemental Material^30,31^). Similar to the UKB, parent-offspring pairs were identified using the KING software,^28^ reported sex, and date of birth.^32^ Only individuals of European ancestry were included in the study (Supplemental Material^33,34^). After cleaning, there were 26 057 mother-offspring pairs and 19 792 father-offspring pairs available for analysis (Table S1).

### Offspring Cardiometabolic Risk Factors

The offspring cardiometabolic risk factors included in our analysis were systolic BP, diastolic BP, body mass index, lipid profile (ie, ApoA [Apolipoprotein A], ApoB [Apolipoprotein B], total cholesterol, LDL-C [low-density lipoprotein cholesterol], Lp(a) [lipoprotein A], HDL-C [high-density lipoprotein cholesterol], and triglycerides), glycemic biomarkers (ie, nonfasting glucose, glycated hemoglobin, and IGF-1 [insulin-like growth factor 1]), and other relevant cardiometabolic traits (ie, CRP [C-reactive protein] and urate). Further details of the collection and availability of UKB and HUNT variables are given in the Supplemental Material^35–41^ and Table S1.

### Selection of BP-Associated single nucleotide polymorphisms (SNPs)

The BP-associated SNPs were identified from external genome-wide association studies performed by the International Blood Pressure Consortium.^35,42–44^ The genome-wide association studies of BP used for the selection of instruments did not include participants from the UKB or HUNT studies in the discovery stages, which avoids potential sample overlap with mothers/fathers that were included in the current analysis. Unweighted genetic scores were constructed by summing BP-raising alleles (Supplemental Material^45,46^ and Table S2).

We conducted 3 analyses to confirm that the BP-associated SNPs from the general population sample also had similar effects on BP during pregnancy (further details are given in the Supplemental Material^22,47^).

### Statistical Analysis

Maternal BP during pregnancy was not physically measured in the UKB or HUNT cohorts; instead, we instrumented this exposure using maternal GRSs for BP. Thus, we directly tested the association between maternal unweighted genetic scores and offspring outcomes in up to 29 708 mother-offspring pairs from UKB and HUNT, adjusting for the offspring’s genetic score calculated from the same BP-associated SNPs (Figure S1B).^32^ The details of the regression analyses and secondary analyses in mother-offspring pairs are given in the Supplemental Material^48,49^ and Table S3.^32,48^

If the effect of maternal BP is operating on offspring via the intrauterine environment, then we would expect no causal relationship in father-offspring pairs. Therefore, we also conducted sensitivity analyses in up to 21 423 father-offspring pairs from the UKB and the HUNT studies to explore the possibility of postnatal effects (Supplemental Material^50^).

We meta-analyzed the results of the primary analyses from the UKB and HUNT studies for each offspring variable using Stouffer Z score which weights each study’s contribution by the square root of the sample size; this facilitated meta-analysis of variables that were scaled differently in UKB versus HUNT.^51^ Meta-analysis was conducted using R (version 3.5.3). In the case of all analyses, we present *P* values that have not been corrected for multiple testing.

### Power Calculation

We calculated the statistical power to detect maternal genetic effects on offspring cardiometabolic risk factors conditional on offspring genotype using the Maternal and Offspring Genetic Effects Power Calculator (https://evansgroup.di.uq.edu.au/MGPC/)^52^ (Supplemental Material).

## Results

### SNPs Associated With BP in Pregnancy

We found strong evidence that our selected BP-associated SNPs from the general population sample have relatively consistent direction of effects on BP during pregnancy and gestational hypertensive disorders in independent cohorts (FinnGen and ALSPAC; Supplemental Material,^53,54^ Figure S2 and Tables S4 and S5).

### Association Between Maternal Genetic Scores and Later-Life Offspring Traits in UKB and HUNT

The results from the analyses assessing the association between unweighted maternal genetic scores for systolic BP- or diastolic BP-associated SNPs and offspring cardiometabolic traits, after adjusting for offspring genetic scores, in the UKB and HUNT studies are presented in the Table, along with the meta-analysis *P* values. We did not detect any association between maternal unweighted genetic scores and cardiometabolic offspring outcomes in the meta-analysis (Table). Similarly, we did not detect any association in the father-offspring pairs in the meta-analysis, consistent with the absence of a postnatal effect operating (Table S6). The results of the main analyses in individual cohorts (UKB and HUNT) are presented in the Table, and the results of sensitivity analyses are given in Tables S7 through S14.

**Table 1.**
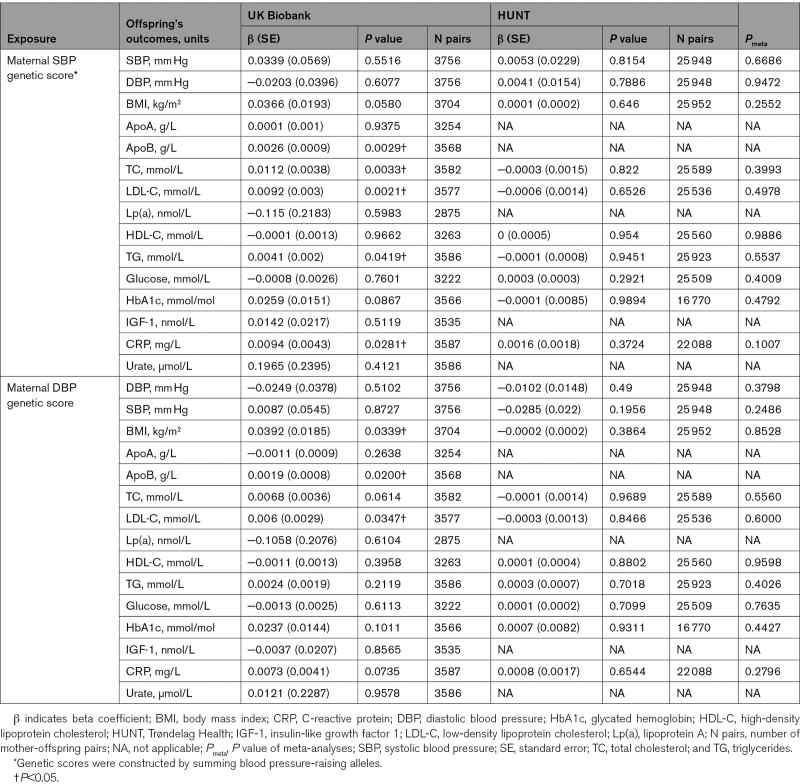
Associations Between the Maternal Genetic Score for Blood Pressure and Offspring’s Traits in UK Biobank and HUNT Studies

### Power Calculations

Power calculations indicated that we had ≥80% power to detect a maternal genetic effect that explained as little as 0.035% of the variance in the offspring cardiometabolic trait with 29 708 mother-offspring pairs (2-tailed α=0.05). For the traits that were available in the UKB only, with 3756 mother-offspring pairs, we were underpowered (19%) to detect an effect size as low as 0.04%; however, we had >80% power to detect a large effect size of 0.28% of the variance in the offspring cardiometabolic outcome (Figures S3 and S4, Table S15, and Supplemental Material).

## Discussion

Our investigation is the largest genetic study to date to have explored the impact of maternal BP during pregnancy on long-term offspring cardiometabolic health. Our study leverages the considerable number of genotyped mother-offspring (and father-offspring) pairs in the UKB and HUNT studies to examine a possible causal relationship between these variables using MR. Importantly, all offspring from the UKB and the majority of offspring from the HUNT study are middle-aged and elderly adults who are old enough to manifest elevated levels of risk factors for cardiometabolic disease. Our results in general, however, did not support a strong association between genetically predicted maternal BP and offspring cardiometabolic risk factors. The implication is that modest increases in maternal BP during pregnancy are unlikely to drive large increases in offspring cardiometabolic risk in later life. This implication is consistent with a previous study of siblings in HUNT.^9^ That study reported that offspring born to hypertensive pregnancies had similar cardiovascular risk factors in young adulthood as their siblings born after normotensive pregnancies, suggesting that the association observed in the unrelated sample was driven by shared genetic or environmental factors, instead of intrauterine effects.

We did not find any strong indications of effects of maternal BP on offspring outcomes, however, in the smaller and underpowered analysis of UKB alone, we did identify 2 nominal associations between maternal systolic BP risk score and ApoB. We were unable to meta-analyze/replicate this finding in the HUNT study as ApoB was not available for analysis. It is also likely given that the UKB analysis on its own is underpowered, that the finding may be due to type 1 error (false positives). Thus, the association needs to be replicated in a larger sample of mother-offspring pairs.

Asymptotic power calculations suggested that our study was well powered (≥80%) to detect an effect size as low as 0.035% of the variance explained in the offspring outcome by the unweighted maternal genetic score. However, given that an unweighted genetic score of BP variants explains about 0.8% in maternal BP, the above power calculation translates to a causal effect of maternal BP on offspring cardiometabolic risk which is quite large (ie, standardized β =0.2). This implies that whilst our study is well powered to rule out strong effects of maternal BP on offspring cardiometabolic risk factors, it has less power to investigate small to moderate effects. The corollary is that the nominal associations found in the UKB are likely to reflect false positives (type 1 error) brought about by multiple testing.

Differences in results between UKB and HUNT may reflect differences in sample size between the studies, and potentially, contrasting selection biases. For example, over 50% of the inhabitants in the Nord-Trøndelag County participated in the HUNT study,^20^ while the UKB study only had a participation rate of 5.45%, tending to enroll healthier people with higher socioeconomic status than the general population.^24,55^

Previous observational association studies in humans^1–4^ have focused on the relationship between gestational hypertension and preeclampsia (ie, gestational hypertension accompanied by maternal organ dysfunction during the second half of pregnancy). We did not specifically investigate gestational hypertension or preeclampsia in the current study due to the lack of genetic variants associated specifically with these diagnoses. A recent genome-wide association study of preeclampsia identified 2 regions of the genome that reached genome-wide significance, both of which have been previously associated with BP in nonpregnant women and men.^56^ Additionally, that study showed that a GRS for hypertension in a sample of nonpregnant women associated with preeclampsia,^56^ providing further evidence for the genetic overlap between the 2 diagnoses. It is, therefore, likely that the GRSs used in our study not only increase maternal BP during pregnancy but also increase risk of preeclampsia.

Our analyses used genetic variants that were associated with BP as a quantitative trait in population-based samples of individuals. We, therefore, did not explicitly model the effect of gestational hypertensive disorders (or preterm births/adverse birth outcomes) in our analyses. However, as GRSs which increase maternal BP are also likely to increase the risk of gestational hypertensive disorders, we expect that the presence of mothers with gestational hypertensive disorders in our data set may also contribute to any association between maternal (BP associated) GRS and future cardiometabolic risk in offspring. Nevertheless, it is difficult to assess the relative contribution of each of these sources of variation to our results without detailed clinical information across pregnancy, with the caveat that our study is likely to be better powered to detect the causal effect of quantitative changes in maternal BP during pregnancy particularly within the normal range (systolic BP<140 mm Hg; diastolic BP<90 mm Hg).^57^ That being said, we note that it is still possible that extreme exposures like gestational hypertension and preeclampsia may causally increase future offspring cardiometabolic risk, but it is difficult to examine these hypotheses via MR until the scientific community discovers genetic instruments that specifically instrument gestational hypertension/preeclampsia.

There are several limitations to the current study. First, our framework does not formally estimate the size of the causal effect of maternal BP on offspring cardiometabolic traits as is done in most MR analyses (ie, because the magnitude of SNP-BP associations may differ in pregnancy compared to in the general population), but it nevertheless uses MR principles to provide evidence for or against a causal relationship between these traits.^14^ Second, we have assumed that genetic variants identified in large genome-wide association studies of BP in males and nonpregnant females are also associated with BP (in a similar direction) in pregnant women. Our analyses performed in pregnant mothers in ALSPAC and FinnGen support the assumption that many BP-associated loci operating in the general population also exert similar effects during pregnancy. Third, we assume a linear relationship between and within maternal BP-associated loci and later-life cardiometabolic traits in their offspring, which may not optimally capture the true relationship between the two. Fourth, the blood tests for lipid and glucose traits were performed using nonfasting samples in both UKB and the HUNT studies which may have influenced the estimates for triglycerides and glucose; however, other biomarkers such as glycated hemoglobin, cholesterol, and lipoprotein levels do not change or only differ minimally in fasting versus nonfasting tests.^58^ Fifth, our model did not completely control for possible pleiotropy through the maternal genome. Although the current model blocks pleiotropic paths through the offspring genome (and addresses the possibility of postnatal pleiotropic effects by performing the same analyses in father-offspring pairs), BP-associated SNPs in the mother could still exert prenatal pleiotropic effects and maternal-specific postnatal effects on offspring cardiometabolic risk through effects other than raising BP. However, this is perhaps less of a concern for the negative results in our study, as any pleiotropic effect would have to have an equal and opposite effect to obscure a true effect of maternal BP on offspring cardiometabolic risk, which is an unlikely scenario. Furthermore, our models do not account for assortative mating, but it seems unlikely that this would cause our observed negative results.^59^ Sixth, we did not have enough power with the current sample size to conduct analyses stratified by offspring sex, to investigate sexual dimorphism in the maternal genetic effects under study. Seventh, because the analyses were conducted only in participants of European descent, the results need to be replicated in other populations. Finally, only a selection of cardiometabolic traits of interest was available in the HUNT study. Therefore, we could not replicate the association between genetically predicted maternal BP and offspring outcomes, such as ApoB and CRP. These associations will need to be replicated in larger cohorts.

## Perspectives

In conclusion, our results suggest that perturbations in maternal BP during pregnancy are unlikely to cause large increases in the risk of offspring cardiometabolic disease in later life. Although previous conventional epidemiological studies have found some evidence for associations between maternal BP and offspring cardiometabolic risk factors, our analyses, which provide a more rigorous assessment of causality, suggest that offspring genetic effects and confounding by environmental factors may be the predominant explanation for such population-level associations. MR studies that specifically examine the long-term effects of extreme exposures like gestational hypertension and preeclampsia on future offspring cardiometabolic risk are needed.

## Article Information

### Acknowledgments

We thank the research participants of the UK Biobank, HUNT (Trøndelag Health), and FinnGen studies and are extremely grateful to all the families who took part in the ALSPAC study (Avon Longitudinal Study of Parents and Children), the midwives for their help in recruiting them, and the whole ALSPAC team, which includes interviewers, computer and laboratory technicians, clerical workers, research scientists, volunteers, managers, receptionists, and nurses. This research has been conducted using the UK Biobank (Reference 12703 and 53641), ALSPAC (Reference B3544) and HUNT resources. The HUNT study is a collaboration between HUNT Research Center (Faculty of Medicine and Health Sciences, Norwegian University of Science and Technology NTNU), Trøndelag County Council, Central Norway Regional Health Authority, and the Norwegian Institute of Public Health.

### Sources of Funding

G. Wang is supported by The University of Queensland Graduate School Scholarship (UQGSS). D.M. Evans is funded by an Australian National Health and Medical Research Council Senior Research Fellowship (APP1137714), and this work was funded by NHMRC project grants (GNT1157714 and GNT1183074). N.M. Warrington is affiliated with a unit that is supported by the UK Medical Research Council (MC_UU_00011/3 and MC_UU_00011/6). J.P. Kemp is funded by a National Health and Medical Research Council (Australia) Investigator grant (GNT1177938). G.-H. Moen is supported by the Norwegian Research Council (Post doctorial mobility research grant 287198), the Norwegian Diabetes Association, Oslo Diabetes Research Centre, and Nils Normans minnegave. T.A. Bond works in/is affiliated with a unit that is supported by the UK Medical Research Council (MC_UU_00011/6) and is supported by the British Heart Foundation Accelerator Award at the University of Bristol (R100643-101). L. Bhatta, B.O. Åsvold, and B. Brumpton receive support from the K.G. Jebsen Center for Genetic Epidemiology funded by Stiftelsen Kristian Gerhard Jebsen; Faculty of Medicine and Health Sciences, NTNU; The Liaison Committee for education, research and innovation in Central Norway; and the Joint Research Committee between St Olavs Hospital and the Faculty of Medicine and Health Sciences, NTNU. The genotyping in HUNT was financed by the National Institute of Health (NIH); University of Michigan; The Research Council of Norway; The Liaison Committee for education, research and innovation in Central Norway; and the Joint Research Committee between St Olavs Hospital and the Faculty of Medicine and Health Sciences, NTNU. The UK Medical Research Council and Wellcome (Grant ref: 217065/Z/19/Z) and the University of Bristol provide core support for ALSPAC (Avon Longitudinal Study of Parents and Children). A comprehensive list of grants funding is available on the ALSPAC website (http://www.bristol.ac.uk/alspac/external/documents/grant-acknowledgements.pdf); this research was specifically funded by Lifelong Health and Wellbeing (LLHW) via the MRC (G1001357), Wellcome Trust (WT092830/Z/10/Z and WT088806), and the British Heart Foundation (SP/07/008/24066). Genome-wide association studies (GWAS) data were generated by Sample Logistics and Genotyping Facilities at Wellcome Sanger Institute and LabCorp (Laboratory Corporation of America) using support from 23andMe. This publication is the work of the authors and G. Wang, L. Bhatta, N.M. Warrington, D.M. Evans, and B. Brumpton will serve as guarantors for the contents of this article.

### Disclosures

None.

## Supplementary Material



## Novelty and Significance

### What Is New?

- Our investigation is the largest genetic study to date to have explored the impact of maternal blood pressure during pregnancy on long-term offspring cardiometabolic health.
- Our analyses used a Mendelian randomization framework to provide a more rigorous assessment of causality.

### What Is Relevant?

- Maternal blood pressure during pregnancy is unlikely to cause large increases in the risk of offspring cardiometabolic diseases in later life, including hypertension.
- Understanding this relationship will help determine whether intervening on high maternal blood pressure during pregnancy will combat the rising incidence of offspring cardiometabolic diseases in adulthood.

### Summary

Our study suggests that high maternal blood pressure, as proxied by maternal SNPs that influence blood pressure is unlikely to be a key determinant of adverse cardiometabolic outcomes in offspring.

## References

[1] Davis EF Lazdam M Lewandowski AJ Worton SA Kelly B Kenworthy Y Adwani S Wilkinson AR McCormick K Sargent I. Cardiovascular risk factors in children and young adults born to preeclamptic pregnancies: a systematic review. Pediatrics. 2012;129:e1552–e1561. doi: 10.1542/peds.2011-3093 22614768 10.1542/peds.2011-3093

[2] Kajantie E Eriksson JG Osmond C Thornburg K Barker DJ. Pre-eclampsia is associated with increased risk of stroke in the adult offspring: the Helsinki birth cohort study. Stroke. 2009;40:1176–1180. doi: 10.1161/STROKEAHA.108.538025 19265049 10.1161/STROKEAHA.108.538025

[3] Jansen MA Pluymen LP Dalmeijer GW Groenhof TKJ Uiterwaal CS Smit HA van Rossem L. Hypertensive disorders of pregnancy and cardiometabolic outcomes in childhood: a systematic review. Eur J Prev Cardiol. 2019;26:1718–1747. doi: 10.1177/2047487319852716 31132891 10.1177/2047487319852716 PMC6806146

[4] Goffin SM Derraik JGB Groom KM Cutfield WS. Maternal pre-eclampsia and long-term offspring health: Is there a shadow cast? Pregnancy Hypertens. 2018;12:11–15. doi: 10.1016/j.preghy.2018.02.003 29674189 10.1016/j.preghy.2018.02.003

[5] Kajantie E Osmond C Eriksson JG. Gestational hypertension is associated with increased risk of type 2 diabetes in adult offspring: the Helsinki Birth Cohort Study. Am J Obstet Gynecol. 2017;216:281.e1–281.e7. doi: 10.1016/j.ajog.2016.10.041 27823919 10.1016/j.ajog.2016.10.041 PMC5503126

[6] Herrera-Garcia G Contag S. Maternal preeclampsia and risk for cardiovascular disease in offspring. Curr Hypertens Rep. 2014;16:475. doi: 10.1007/s11906-014-0475-3 25097112 10.1007/s11906-014-0475-3

[7] Andraweera PH Lassi ZS. Cardiovascular risk factors in offspring of preeclamptic pregnancies-systematic review and meta-analysis. J Pediatr. 2019;208:104–113.e6. doi: 10.1016/j.jpeds.2018.12.008 30876753 10.1016/j.jpeds.2018.12.008

[8] Warrington NM Beaumont RN Horikoshi M Day FR Helgeland Ø Laurin C Bacelis J Peng S Hao K Feenstra B; EGG Consortium. Maternal and fetal genetic effects on birth weight and their relevance to cardio-metabolic risk factors. Nat Genet. 2019;51:804–814. doi: 10.1038/s41588-019-0403-1 31043758 10.1038/s41588-019-0403-1 PMC6522365

[9] Alsnes IV Vatten LJ Fraser A Bjørngaard JH Rich-Edwards J Romundstad PR Åsvold BO. Hypertension in pregnancy and offspring cardiovascular risk in young adulthood: prospective and sibling studies in the HUNT Study (Nord-Trøndelag Health Study) in Norway. Hypertension. 2017;69:591–598. doi: 10.1161/HYPERTENSIONAHA.116.08414 28223467 10.1161/HYPERTENSIONAHA.116.08414

[10] Kurbasic A Fraser A Mogren I Hallmans G Franks PW Rich-Edwards JW Timpka S. Maternal hypertensive disorders of pregnancy and offspring risk of hypertension: A Population-Based Cohort and Sibling Study. Am J Hypertens. 2019;32:331–334. doi: 10.1093/ajh/hpy176 30475953 10.1093/ajh/hpy176 PMC6420682

[11] Lawlor DA Harbord RM Sterne JA Timpson N Davey Smith G. Mendelian randomization: using genes as instruments for making causal inferences in epidemiology. Stat Med. 2008;27:1133–1163. doi: 10.1002/sim.3034 17886233 10.1002/sim.3034

[12] Smith GD Ebrahim S. ‘Mendelian randomization’: can genetic epidemiology contribute to understanding environmental determinants of disease? Int J Epidemiol. 2003;32:1–22. doi: 10.1093/ije/dyg070 12689998 10.1093/ije/dyg070

[13] Smith GD Lawlor DA Harbord R Timpson N Day I Ebrahim S. Clustered environments and randomized genes: a fundamental distinction between conventional and genetic epidemiology. PLoS Med. 2007;4:e352. doi: 10.1371/journal.pmed.0040352 18076282 10.1371/journal.pmed.0040352 PMC2121108

[14] Evans DM Moen GH Hwang LD Lawlor DA Warrington NM. Elucidating the role of maternal environmental exposures on offspring health and disease using two-sample Mendelian randomization. Int J Epidemiol. 2019;48:861–875. doi: 10.1093/ije/dyz019 30815700 10.1093/ije/dyz019 PMC6659380

[15] Birth-Gene Study Working Group. Huang T Wang T Zheng Y Ellervik C Li X Gao M Fang Z Chai JF Ahluwalia TVS Wang Y. Birth-Gene Study Working Group. Association of birth weight with type 2 diabetes and glycemic traits: A Mendelian Randomization Study. JAMA Netw Open. 2019;2:e1910915. doi: 10.1001/jamanetworkopen.2019.10915 31539074 10.1001/jamanetworkopen.2019.10915 PMC6755534

[16] Zanetti D Tikkanen E Gustafsson S Priest JR Burgess S Ingelsson E. Birthweight, type 2 diabetes mellitus, and cardiovascular disease: addressing the barker hypothesis with mendelian randomization. Circ Genom Precis Med. 2018;11:e002054. doi: 10.1161/CIRCGEN.117.002054 29875125 10.1161/CIRCGEN.117.002054 PMC6447084

[17] Wang T Huang T Li Y Zheng Y Manson JE Hu FB Qi L. Low birthweight and risk of type 2 diabetes: a Mendelian randomisation study. Diabetologia. 2016;59:1920–1927. doi: 10.1007/s00125-016-4019-z 27333884 10.1007/s00125-016-4019-z PMC4970938

[18] D’Urso S Wang G Hwang LD Moen GH Warrington NM Evans DM. A cautionary note on using Mendelian randomization to examine the Barker hypothesis and Developmental Origins of Health and Disease (DOHaD). J Dev Orig Health Dis. 2020;12:688–693. doi: 10.1017/S2040174420001105 33272351 10.1017/S2040174420001105

[19] Sudlow C Gallacher J Allen N Beral V Burton P Danesh J Downey P Elliott P Green J Landray M. UK biobank: an open access resource for identifying the causes of a wide range of complex diseases of middle and old age. PLoS Med. 2015;12:e1001779. doi: 10.1371/journal.pmed.1001779 25826379 10.1371/journal.pmed.1001779 PMC4380465

[20] Krokstad S Langhammer A Hveem K Holmen TL Midthjell K Stene TR Bratberg G Heggland J Holmen J. Cohort profile: the HUNT Study, Norway. Int J Epidemiol. 2013;42:968–977. doi: 10.1093/ije/dys095 22879362 10.1093/ije/dys095

[21] Boyd A Golding J Macleod J Lawlor DA Fraser A Henderson J Molloy L Ness A Ring S Davey Smith G. Cohort profile: the ‘children of the 90s’–the index offspring of the Avon Longitudinal Study of Parents and Children. Int J Epidemiol. 2013;42:111–127. doi: 10.1093/ije/dys064 22507743 10.1093/ije/dys064 PMC3600618

[22] Fraser A Macdonald-Wallis C Tilling K Boyd A Golding J Davey Smith G Henderson J Macleod J Molloy L Ness A. Cohort profile: the Avon Longitudinal Study of Parents and Children: ALSPAC mothers cohort. Int J Epidemiol. 2013;42:97–110. doi: 10.1093/ije/dys066 22507742 10.1093/ije/dys066 PMC3600619

[23] Harris PA Taylor R Thielke R Payne J Gonzalez N Conde JG. Research electronic data capture (REDCap)–a metadata-driven methodology and workflow process for providing translational research informatics support. J Biomed Inform. 2009;42:377–381. doi: 10.1016/j.jbi.2008.08.010 18929686 10.1016/j.jbi.2008.08.010 PMC2700030

[24] Fry A Littlejohns TJ Sudlow C Doherty N Adamska L Sprosen T Collins R Allen NE. Comparison of sociodemographic and health-related characteristics of UK biobank participants with those of the general population. Am J Epidemiol. 2017;186:1026–1034. doi: 10.1093/aje/kwx246 28641372 10.1093/aje/kwx246 PMC5860371

[25] Bycroft C Freeman C Petkova D Band G Elliott LT Sharp K Motyer A Vukcevic D Delaneau O O’Connell J. The UK biobank resource with deep phenotyping and genomic data. Nature. 2018;562:203–209. doi: 10.1038/s41586-018-0579-z 30305743 10.1038/s41586-018-0579-z PMC6786975

[26] 1000 Genomes Project Consortium. Auton A Brooks LD Durbin RM Garrison EP Kang HM Korbel JO Marchini JL McCarthy S McVean GA Abecasis GR. 1000 Genomes Project Consortium. A global reference for human genetic variation. Nature. 2015;526:68–74. doi: 10.1038/nature15393 26432245 10.1038/nature15393 PMC4750478

[27] Abraham G Qiu Y Inouye M. FlashPCA2: principal component analysis of Biobank-scale genotype datasets. Bioinformatics. 2017;33:2776–2778. doi: 10.1093/bioinformatics/btx299 28475694 10.1093/bioinformatics/btx299

[28] Manichaikul A Mychaleckyj JC Rich SS Daly K Sale M Chen WM. Robust relationship inference in genome-wide association studies. Bioinformatics. 2010;26:2867–2873. doi: 10.1093/bioinformatics/btq559 20926424 10.1093/bioinformatics/btq559 PMC3025716

[29] Holmen TL Bratberg G Krokstad S Langhammer A Hveem K Midthjell K Heggland J Holmen J. Cohort profile of the Young-HUNT Study, Norway: a population-based study of adolescents. Int J Epidemiol. 2014;43:536–544. doi: 10.1093/ije/dys232 23382364 10.1093/ije/dys232

[30] Ferreira MA Vonk JM Baurecht H Marenholz I Tian C Hoffman JD Helmer Q Tillander A Ullemar V van Dongen J; 23andMe Research Team; AAGC collaborators; BIOS consortium; LifeLines Cohort Study. Shared genetic origin of asthma, hay fever and eczema elucidates allergic disease biology. Nat Genet. 2017;49:1752–1757. doi: 10.1038/ng.3985 29083406 10.1038/ng.3985 PMC5989923

[31] Friedewald WT Levy RI Fredrickson DS. Estimation of the concentration of low-density lipoprotein cholesterol in plasma, without use of the preparative ultracentrifuge. Clin Chem. 1972;18:499–502. doi: 10.1093/clinchem/18.6.499 4337382

[32] Moen GH Brumpton B Willer C Åsvold BO Birkeland KI Wang G Neale MC Freathy RM Smith GD Lawlor DA. Mendelian randomization study of maternal influences on birthweight and future cardiometabolic risk in the HUNT cohort. Nat Commun. 2020;11:5404. doi: 10.1038/s41467-020-19257-z 33106479 10.1038/s41467-020-19257-z PMC7588432

[33] Wang C Zhan X Bragg-Gresham J Kang HM Stambolian D Chew EY Branham KE Heckenlively J Fulton R Wilson RK; FUSION Study. Ancestry estimation and control of population stratification for sequence-based association studies. Nat Genet. 2014;46:409–415. doi: 10.1038/ng.2924 24633160 10.1038/ng.2924 PMC4084909

[34] Li JZ Absher DM Tang H Southwick AM Casto AM Ramachandran S Cann HM Barsh GS Feldman M Cavalli-Sforza LL. Worldwide human relationships inferred from genome-wide patterns of variation. Science. 2008;319:1100–1104. doi: 10.1126/science.1153717 18292342 10.1126/science.1153717

[35] International Consortium for Blood Pressure Genome-Wide Association Studies. Ehret GB Munroe PB Rice KM Bochud M Johnson AD Chasman DI Smith AV Tobin MD Verwoert GC Hwang S-J. International Consortium for Blood Pressure Genome-Wide Association Studies. Genetic variants in novel pathways influence blood pressure and cardiovascular disease risk. Nature. 2011;478:103–109. doi: 10.1038/nature10405 21909115 10.1038/nature10405 PMC3340926

[36] Tobin MD Sheehan NA Scurrah KJ Burton PR. Adjusting for treatment effects in studies of quantitative traits: antihypertensive therapy and systolic blood pressure. Stat Med. 2005;24:2911–2935. doi: 10.1002/sim.2165 16152135 10.1002/sim.2165

[37] Boekholdt SM Arsenault BJ Mora S Pedersen TR LaRosa JC Nestel PJ Simes RJ Durrington P Hitman GA Welch KM. Association of LDL cholesterol, non-HDL cholesterol, and apolipoprotein B levels with risk of cardiovascular events among patients treated with statins: a meta-analysis. JAMA. 2012;307:1302–1309. doi: 10.1001/jama.2012.366 22453571 10.1001/jama.2012.366

[38] Locke AE Steinberg KM Chiang CWK Service SK Havulinna AS Stell L Pirinen M Abel HJ Chiang CC Fulton RS; FinnGen Project. Exome sequencing of Finnish isolates enhances rare-variant association power. Nature. 2019;572:323–328. doi: 10.1038/s41586-019-1457-z 31367044 10.1038/s41586-019-1457-z PMC6697530

[39] Liu DJ Peloso GM Yu H Butterworth AS Wang X Mahajan A Saleheen D Emdin C Alam D Alves AC; Charge Diabetes Working Group; EPIC-InterAct Consortium; EPIC-CVD Consortium; GOLD Consortium; VA Million Veteran Program. Exome-wide association study of plasma lipids in >300,000 individuals. Nat Genet. 2017;49:1758–1766. doi: 10.1038/ng.3977 29083408 10.1038/ng.3977 PMC5709146

[40] Asselbergs FW Guo Y van Iperen EP Sivapalaratnam S Tragante V Lanktree MB Lange LA Almoguera B Appelman YE Barnard J; LifeLines Cohort Study. Large-scale gene-centric meta-analysis across 32 studies identifies multiple lipid loci. Am J Hum Genet. 2012;91:823–838. doi: 10.1016/j.ajhg.2012.08.032 23063622 10.1016/j.ajhg.2012.08.032 PMC3487124

[41] Horikoshi M Beaumont RN Day FR Warrington NM Kooijman MN Fernandez-Tajes J Feenstra B van Zuydam NR Gaulton KJ Grarup N; CHARGE Consortium Hematology Working Group; Early Growth Genetics (EGG) Consortium. Genome-wide associations for birth weight and correlations with adult disease. Nature. 2016;538:248–252. doi: 10.1038/nature19806 27680694 10.1038/nature19806 PMC5164934

[42] Ehret GB Ferreira T Chasman DI Jackson AU Schmidt EM Johnson T Thorleifsson G Luan J Donnelly LA Kanoni S; CHARGE-EchoGen consortium; CHARGE-HF consortium; Wellcome Trust Case Control Consortium. The genetics of blood pressure regulation and its target organs from association studies in 342,415 individuals. Nat Genet. 2016;48:1171–1184. doi: 10.1038/ng.3667 27618452 10.1038/ng.3667 PMC5042863

[43] Surendran P Drenos F Young R Warren H Cook JP Manning AK Grarup N Sim X Barnes DR Witkowska K; CHARGE-Heart Failure Consortium; EchoGen Consortium; METASTROKE Consortium; GIANT Consortium; EPIC-InterAct Consortium; Lifelines Cohort Study; Wellcome Trust Case Control Consortium; Understanding Society Scientific Group; EPIC-CVD Consortium; CHARGE+ Exome Chip Blood Pressure Consortium; T2D-GENES Consortium; GoT2DGenes Consortium; ExomeBP Consortium; CHD Exome+ Consortium. Trans-ancestry meta-analyses identify rare and common variants associated with blood pressure and hypertension. Nat Genet. 2016;48:1151–1161. doi: 10.1038/ng.3654 27618447 10.1038/ng.3654 PMC5056636

[44] Warren HR Evangelou E Cabrera CP Gao H Ren M Mifsud B Ntalla I Surendran P Liu C Cook JP; International Consortium of Blood Pressure (ICBP) 1000G Analyses; BIOS Consortium; Lifelines Cohort Study; Understanding Society Scientific group; CHD Exome+ Consortium; ExomeBP Consortium; T2D-GENES Consortium; GoT2DGenes Consortium; Cohorts for Heart and Ageing Research in Genome Epidemiology (CHARGE) BP Exome Consortium; International Genomics of Blood Pressure (iGEN-BP) Consortium; UK Biobank CardioMetabolic Consortium BP working group. Genome-wide association analysis identifies novel blood pressure loci and offers biological insights into cardiovascular risk. Nat Genet. 2017;49:403–415. doi: 10.1038/ng.376828135244

[45] Chang CC Chow CC Tellier LC Vattikuti S Purcell SM Lee JJ. Second-generation PLINK: rising to the challenge of larger and richer datasets. Gigascience. 2015;4:7. doi: 10.1186/s13742-015-0047-8 25722852 10.1186/s13742-015-0047-8 PMC4342193

[46] Kraft P. Curses–winner’s and otherwise–in genetic epidemiology. Epidemiology. 2008;19:649–657. doi: 10.1097/EDE.0b013e318181b865 18703928 10.1097/EDE.0b013e318181b865

[47] Cuellar-Partida G Lundberg M Kho PF D’Urso S Gutierrez-Mondragon LF Hwang L-D. Complex-traits genetics virtual lab: a community-driven web platform for post-GWAS analyses. bioRxiv. Preprint posted online May 9, 2019. doi: 10.1101/518027

[48] Boker S Neale M Maes H Wilde M Spiegel M Brick T Spies J Estabrook R Kenny S Bates T. OpenMx: an open source extended structural equation modeling framework. Psychometrika. 2011;76:306–317. doi: 10.1007/s11336-010-9200-6 23258944 10.1007/s11336-010-9200-6 PMC3525063

[49] Evangelou E Warren HR Mosen-Ansorena D Mifsud B Pazoki R Gao H Ntritsos G Dimou N Cabrera CP Karaman I; Million Veteran Program. Genetic analysis of over 1 million people identifies 535 new loci associated with blood pressure traits. Nat Genet. 2018;50:1412–1425. doi: 10.1038/s41588-018-0205-x 30224653 10.1038/s41588-018-0205-x PMC6284793

[50] Lane M Robker RL Robertson SA. Parenting from before conception. Science. 2014;345:756–760. doi: 10.1126/science.1254400 25124428 10.1126/science.1254400

[51] de Bakker PI Ferreira MA Jia X Neale BM Raychaudhuri S Voight BF. Practical aspects of imputation-driven meta-analysis of genome-wide association studies. Hum Mol Genet. 2008;17(R2):R122–R128. doi: 10.1093/hmg/ddn288 18852200 10.1093/hmg/ddn288 PMC2782358

[52] Moen GH Hemani G Warrington NM Evans DM. Calculating power to detect maternal and offspring genetic effects in genetic association studies. Behav Genet. 2019;49:327–339. doi: 10.1007/s10519-018-9944-9 30600410 10.1007/s10519-018-9944-9

[53] Bulik-Sullivan B Finucane HK Anttila V Gusev A Day FR Loh PR Duncan L Perry JR Patterson N Robinson EB; ReproGen Consortium; Psychiatric Genomics Consortium; Genetic Consortium for Anorexia Nervosa of the Wellcome Trust Case Control Consortium 3. An atlas of genetic correlations across human diseases and traits. Nat Genet. 2015;47:1236–1241. doi: 10.1038/ng.3406 26414676 10.1038/ng.3406 PMC4797329

[54] Bulik-Sullivan BK Loh PR Finucane HK Ripke S Yang J Patterson N Daly MJ Price AL Neale BM; Schizophrenia Working Group of the Psychiatric Genomics Consortium. LD Score regression distinguishes confounding from polygenicity in genome-wide association studies. Nat Genet. 2015;47:291–295. doi: 10.1038/ng.3211 25642630 10.1038/ng.3211 PMC4495769

[55] Munafò MR Tilling K Taylor AE Evans DM Davey Smith G. Collider scope: when selection bias can substantially influence observed associations. Int J Epidemiol. 2018;47:226–235. doi: 10.1093/ije/dyx206 29040562 10.1093/ije/dyx206 PMC5837306

[56] Steinthorsdottir V McGinnis R Williams NO Stefansdottir L Thorleifsson G Shooter S Fadista J Sigurdsson JK Auro KM Berezina G; FINNPEC Consortium; GOPEC Consortium. Genetic predisposition to hypertension is associated with preeclampsia in European and Central Asian women. Nat Commun. 2020;11:5976. doi: 10.1038/s41467-020-19733-6 33239696 10.1038/s41467-020-19733-6 PMC7688949

[57] Williams B Mancia G Spiering W Agabiti Rosei E Azizi M Burnier M Clement DL Coca A de Simone G Dominiczak A; Authors/Task Force Members. 2018 ESC/ESH Guidelines for the management of arterial hypertension: The Task Force for the management of arterial hypertension of the European Society of Cardiology and the European Society of Hypertension: The Task Force for the management of arterial hypertension of the European Society of Cardiology and the European Society of Hypertension. J Hypertens. 2018;36:1953–2041. doi: 10.1097/HJH.0000000000001940 30234752 10.1097/HJH.0000000000001940

[58] Langsted A Nordestgaard BG. Nonfasting versus fasting lipid profile for cardiovascular risk prediction. Pathology. 2019;51:131–141. doi: 10.1016/j.pathol.2018.09.062 30522787 10.1016/j.pathol.2018.09.062

[59] Brumpton B Sanderson E Heilbron K Hartwig FP Harrison S Vie GÅ Cho Y Howe LD Hughes A Boomsma DI; Within-family Consortium; 23andMe Research Team. Avoiding dynastic, assortative mating, and population stratification biases in Mendelian randomization through within-family analyses. Nat Commun. 2020;11:3519. doi: 10.1038/s41467-020-17117-4 32665587 10.1038/s41467-020-17117-4 PMC7360778

