# Supplementary figures and images for: Investigating a Potential Causal Relationship Between Maternal Blood Pressure During Pregnancy and Future Offspring Cardiometabolic Health
Source: Hypertension. 2021 Nov 17;79(1):170–7. doi: 10.1161/HYPERTENSIONAHA.121.17701 (PMC8654122; doi:10.1161/HYPERTENSIONAHA.121.17701)

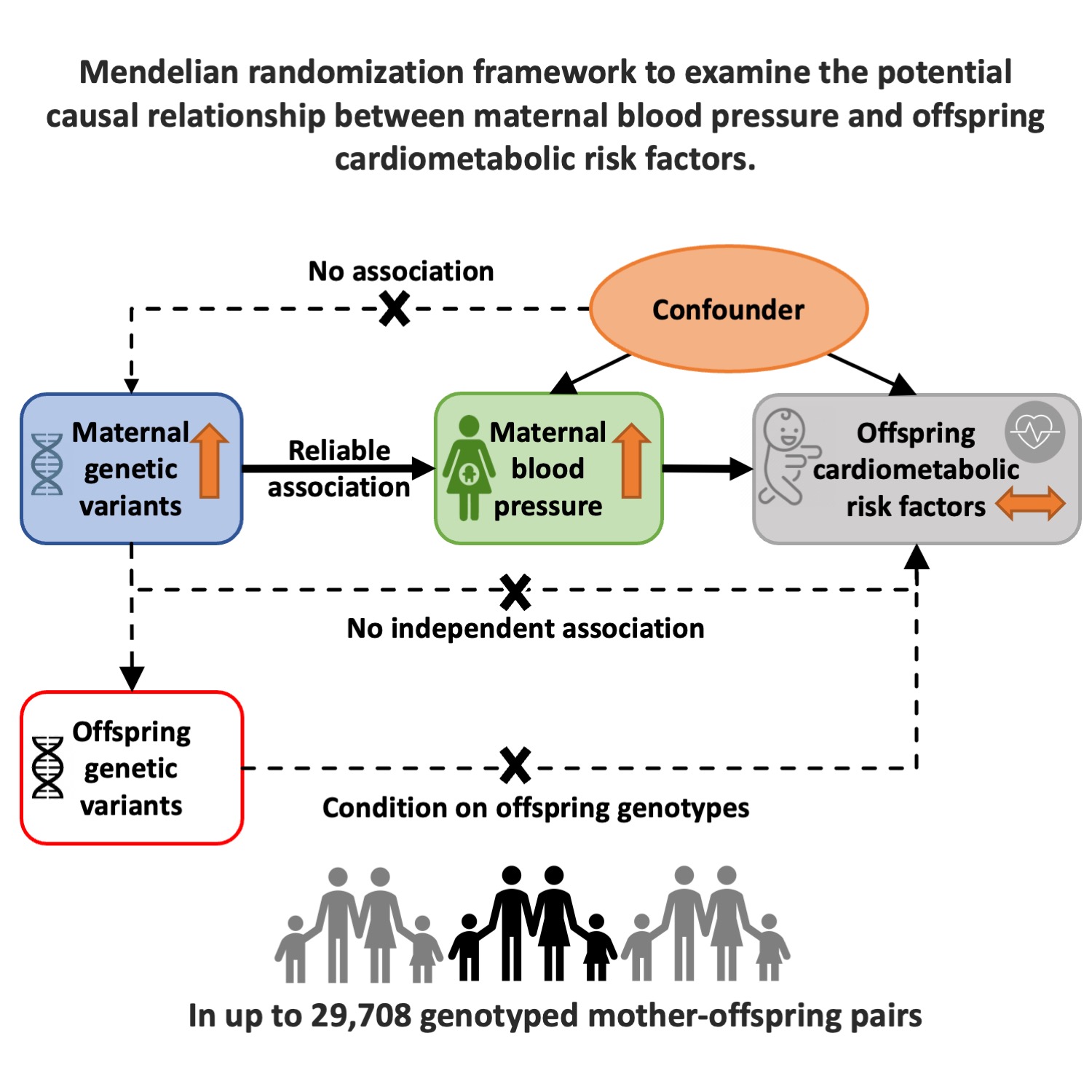

Supplement: Supplementary file 1 [file hyp-79-170-s001.jpg]
