# Supplementary material for: Investigating a Potential Causal Relationship Between Maternal Blood Pressure During Pregnancy and Future Offspring Cardiometabolic Health
Source: Hypertension. 2021 Nov 17;79(1):170–7. doi: 10.1161/HYPERTENSIONAHA.121.17701 (PMC8654122; doi:10.1161/HYPERTENSIONAHA.121.17701)
Supplement: Supplementary file 3 [file hyp-79-170-s003.docx]

Investigating a potential causal relationship between maternal blood pressure during pregnancy and future offspring cardiometabolic health.

Geng Wang ^1, #^, Laxmi Bhatta ^2, #^, Gunn-Helen Moen ^1, 2, 3, 4^ , Liang-Dar Hwang ^1,5^, John P Kemp ^1, 5,6^, Tom A Bond ^1, 4, 6^ , Bjørn Olav Åsvold ^2, 7, 9^, Ben Brumpton ^2, 8, 9*^, David M Evans ^1, 5, 6*^, Nicole M Warrington ^1, 2, 5, 6*^

1. The University of Queensland Diamantina Institute, The University of Queensland, Brisbane, Australia.

2. K.G. Jebsen Center for Genetic Epidemiology, Department of Public Health and Nursing, NTNU, Norwegian University of Science and Technology, Trondheim, Norway.

3. Institute of Clinical Medicine, Faculty of Medicine, University of Oslo, Oslo, Norway.

4. Population Health Sciences, Bristol Medical School, University of Bristol, Bristol, UK.

5. Institute of Molecular Biosciences, The University of Queensland, Brisbane, Australia.

6. Medical Research Council Integrative Epidemiology Unit, University of Bristol, UK.

7. Department of Endocrinology, Clinic of Medicine, St. Olavs Hospital, Trondheim University Hospital, Trondheim, Norway.

8. Clinic of Medicine, St. Olavs Hospital, Trondheim University Hospital, Trondheim, Norway.

9. HUNT Research Center, Department of Public Health and Nursing, NTNU, Norwegian University of Science and Technology, Levanger, Norway.

# These authors contributed equally to this work

* These authors jointly supervised this work

Short title: Maternal blood pressure and offspring health

Corresponding author:

Dr Nicole M Warrington

Research Fellow

The University of Queensland Diamantina Institute

Address:

Level 5, 37 Kent St

Translational Research Institute (TRI)

Woolloongabba, QLD 4102, Australia.

Phone: +61 7 3443 7347

Fax: +61 7 3443 6966

Email: [n.warrington@uq.edu.au](mailto:n.warrington@uq.edu.au)

**Supplementary Materials**

**Supplementary Text**

**Cohort profiles of UK Biobank study (UKB), The Trøndelag Health study (HUNT), and the Avon Longitudinal Study of Parents and Children (ALSPAC)**

The UKB Study is a study of over 500,000 volunteers (with 5.45% response rate of those invited ^1^), recruited from across the UK at age 40-69 years between 2006 and 2010, with a broad range of health-related information and genome-wide genetic data ^2^. Genotyping, quality control, and imputation were performed centrally by the UKB^2^. The UKB samples was genotyped using one of two different Affymetrix arrays (the UK BiLEVE Axiom array or the UK Biobank Axiom array). We utilized imputed genetic data from the May 2017 release from the UKB for our analysis. The UK Biobank has approval from the North West Multi-Centre Research Ethics Committee, which covers the United Kingdom. Participants of all studies provided written informed consent.

The HUNT study invited the entire adult population (≥20 years) of northern Trøndelag to attend clinical examinations and answer questionnaires. Detailed phenotyping has been performed in four study rounds so far, including HUNT1 (1984 to 1986, N=75,027, 86.8% of invited), HUNT2 (1995 to 1997, N=65,402, 69.7% of invited), HUNT3 (2006 to 2008, N=50,663, 54.0% of invited), and HUNT4 (2017 to 2019, N=56,042, 54.0% of invited). About 90% of participants from HUNT2 and HUNT3 were genotyped by genome-wide SNP array in 2015, which was used in the current analysis ^3^. The genotyping and quality control metrics were performed centrally by the HUNT study. HUNT samples were genotyped using one of three different Illumina HumanCoreExome arrays (HumanCoreExome12 v1.0, HumanCoreExome12 v1.1 and UM HUNT Biobank v1.0). Quality control of the genetic data, ancestry and relatedness identification, and the extraction of outcome variables are described in detail elsewhere ^4^. The HUNT Study was approved by the Regional Committee for Medical and Health Research Ethics, Norway and all participants gave written informed consent.

ALSPAC is a population based prospective birth cohort^5,6^. Pregnant women resident in Avon, UK with expected dates of delivery 1st April 1991 to 31st December 1992 were invited to take part in the study. The initial number of pregnancies enrolled is 14,541 (for these at least one questionnaire has been returned or a “Children in Focus” clinic had been attended by 19/07/99). Of these initial pregnancies, there was a total of 14,676 foetuses, resulting in 14,062 live births and 13,988 children who were alive at 1 year of age. Study data were collected and managed using REDCap electronic data capture tools hosted at the University of Bristol ^7^. The study website contains details of all the data that is available through a fully searchable data dictionary and variable search tool: <http://www.bristol.ac.uk/alspac/researchers/our-data/>. Ethical approval for the study was obtained from the ALSPAC Ethics and Law Committee and the Local Research Ethics Committees. Informed consent for the use of data collected via questionnaires and clinics was obtained from participants following the recommendations of the ALSPAC Ethics and Law Committee at the time.

**Ancestry identification in UKB and HUNT studies**

We defined a subset of participants of “European” origin by conducting an ancestry informative principal components (PC) analysis using participants from Phase 3 of the 1000 Genomes project ^8^ as a reference for ancestry. We defined a subset of participants of “European” origin by conducting an ancestry informative principal components (PC) analysis using participants from Phase 3 of the 1000 Genomes project ^8^ as a reference for ancestry. Directly genotyped data was used for ethnic ancestry. The UKB participants were then projected into this PC space according to the SNP loadings generated from the 1000 Genomes PC analysis using FlashPCA2^9^. PC1, PC2 and PC5 resolved the British/European (GBR/CEU [i.e. British in England and Scotland/Western European Ancestry] cluster efficiently and were hence used in subsequent clustering. The UKB participants’ ancestry was classified using an Expectation Maximization Clustering (EMC) algorithm (<https://CRAN.R-project.org/package=EMC> ) centred on the 26 different 1000 genomes populations. After comparing how well the EMC clustering model fit the data using the chosen PCs and by varying the numbers of predefined clusters (1-50 cluster), 12 clusters showed an optimal balance between improved model fit and resolution. Those UKB participants clustering with the GBR/CEU clusters were classified as having “white British” ancestry.

In HUNT, participants of “European” origin were previously defined by conducting an ancestry informative principal components (PC) analysis using participants from the Human Genome Diversity Project (HGDP) reference panel ^10,11^.

**Kinship estimation in UKB**

Parent-offspring pairs were identified using the software defaults for the estimated kinship coefficients and IBS0 cut-offs^2,12^. For each parent-offspring pair in UKB, parent and offspring status was determined using reported sex and date of birth. Offspring who self-reported being part of a multiple birth were excluded from analyses (67 mother-offspring pairs and 20 father-offspring pairs). For parents who had multiple offspring in the dataset, only the eldest offspring with available data was included in the analysis. We further excluded one of any related pair of mothers or fathers in the remaining pairs (unrelated pairs were those that were > 3^rd^-degree relatives defined by KING software, i.e. kinship coefficient > $\frac{1}{2^{9/2}}$ $\frac{1}{2^{9/2}}$) ^12^.

**Outcome measurements and phenotypic quality control in UKB and HUNT studies**

UKB participants had their SBP and DBP measured at three time points (baseline [initial assessment visit], and two follow-ups [first repeat assessment visit and imaging visit]), using either an automated machine (Omron 705 IT electronic blood pressure monitor) or manually using a sphygmomanometer using standard procedures (<https://biobank.ndph.ox.ac.uk/showcase/refer.cgi?id=100225> ). Each participant had their blood pressure measurement taken twice a few moments apart at each follow-up. Two valid readings were available for most participants (> 97%). For each follow-up, the average of the two SBP/DBP measures was calculated if both were recorded; otherwise, only one recorded measure was used. Participants whose two readings were more than 4.56 standard deviations apart were excluded from analyses. If a participant reported being on blood pressure lowering medication at the time of their measurement, then 15mmHg and 10 mmHg were added to their SBP and DBP measurements, respectively^13^. We chose this procedure over including medication use as a covariate as the latter has been shown to lead to biased estimates of the true causal effect ^14^. If participants had an SBP/DBP measurement from the automated machine at baseline, then this measure was used for analysis; otherwise, their (automated) SBP/DBP measurements from follow-up one or two, or their manual measurements from baseline, follow-up one or follow-up two were used (in this order).

UKB participants provided a non-fasting blood sample at two time points (baseline and one follow-up), which were assayed for a panel of biomarkers using standard laboratory procedures ([http://Biobank.ndph.ox.ac.uk/showcase/refer.cgi?id=5636](http://biobank.ndph.ox.ac.uk/showcase/refer.cgi?id=5636)). Total cholesterol, LDL, HDL, TG, ApoA, ApoB, Glucose, urate and CRP were measured by Beckman Coulter AU5800 Clinical Chemistry Analyzer (Beckman Coulter reagents). Total cholesterol and TG were measured using enzymatic (CHOD-POD) method. Direct LDL was measured by using enzymatic selective protection method. HDL was measured by using enzyme immunoinhibition method. ApoA, ApoB, and CRP immunoturbidimetric method. Urate was measured using Uricase-PAP method. Lipoprotein(a) was measured using immunoturbidimetric method by Beckman Coulter AU5800 Clinical Chemistry Analyzer (Randox reagent). IGF-1 was measured using Chemiluminescence Enzyme Immunoassays (CLIA) method by LIASON XL (Serum DiaSorin Ltd). HbA1c was measured using high-performance liquid chromatography (HPLC) method by VARIANT II TURBO Hemoglobin Testing System (Bio-Rad reagent). If the participant reported being on cholesterol-lowering medication (3.5% of participants), LDL-C, total cholesterol, TG, and ApoB were adjusted by dividing by the constants 0.7, 0.8, 0.8, and 0.75 respectively^15-18^. If the participant reported being on insulin (0.6% of participants) at the time of their sample, they were excluded from the analyses of glycaemic biomarkers. If participants had a test result at baseline, then this was used, otherwise their test result at follow-up was used. TG and CRP were natural log-transformed prior to analyses. Standing height and weight were measured in cm and kg, respectively, at two time points (baseline and one follow-up). BMI was then calculated using the formula weight (kg)/height^2^ (m^2^). If participants had a BMI measurement at baseline, then this was used. Otherwise, their BMI at follow-up was used. Finally, all measures of each trait greater or less than 4.56 standard deviations from the mean were set to missing^19^. In UKB, information on each outcome variable was available in 69.8% to 91.2% of mother-offspring pairs (i.e. N of offspring had phenotypic data available / N of all mother-offspring pairs).

We included a subset of the cardiometabolic variables that were available in the HUNT study (HUNT2-HUNT4), including SBP, DBP, BMI, LDL-C, HDL-C, total cholesterol, TG, non-fasting glucose, HbA1c and CRP (Table S1). The remaining outcomes analysed in UKB were not currently available in the HUNT study (i.e. ApoA, ApoB, Lp(a), IGF1 and urate).

In HUNT2 and HUNT3, the SBP and DBP were measured using an automated measure based on oscillometry using Dinamap 8100/845XT (Critikon; <https://hunt-db.medisin.ntnu.no/hunt-db/instrument/DMap> ). For each participant, 2 or 3 measurements of SBP and DBP were taken 1 minute apart. If 3 measurements were available, then the average of last 2 were calculated but if 2 measurements were available then the last reading was used for the analysis. Participants whose two readings were more than 4.56 standard deviations apart were excluded from analyses. The blood pressure measurements for participants on blood pressure increasing medication were increased by 15mmHg and 10mmHg for SBP and DBP, respectively, consistent with the UKB analyses. For the blood measurements, samples were taken from non-fasting participants, which were assayed for a panel of biomarkers using standard laboratory procedures (<https://hunt-db.medisin.ntnu.no/hunt-db/variablelist>). Glucose, TG, TC, and HDL-C were measured in non-fasting serum using Hitachi 911 Autoanalyzer in HUNT2 and Architect ci8200 in HUNT3. Glucose was measured using an enzymatic hexokinase method in HUNT2 and hexokinase/G-6-PDH method in HUNT3. TG was measured using an enzymatic coulometric method in HUNT2 and glycerol phosphate oxidase method in HUNT3. TC was measured using enzymatic cholesterol esterase method in HUNT2 and HUNT3. HDL-C was measured using enzymatic cholesterol oxidase method in HUNT2 and accelerator selective detergent method in HUNT3. Non-fasting LDL cholesterol was calculated using the Friedewald formula ^20^. In a non-fasting whole blood sample, HbA1c was measured using enzymatic HbA1c assay method and Architect ci8200 instrument. It was not available in HUNT2 and HUNT3, therefore the HbA1c available at HUNT4 for those who also participate in HUNT2 or HUNT3 were used. Non-fasting serum CRP was only measured in HUNT3 and HUNT4 using latex immunoassay method and Architect ci8200 instrument. The detection limit of CRP was 0.1 to 160 mg/L where the measurements below or above this range were recorded as 0.05 and 160.1 mg/L, respectively. If CRP was measured in HUNT4, then it was used in the study, otherwise, CRP from HUNT3 were used. For the rest of measurements, if measured variables from HUNT3 were available, then these were used in the study, otherwise, measurements from HUNT2 were used. BMI was calculated as measured weight divided by the squared value of measured height. HbA1c was only available in HUNT4 study round. CRP was only measured in HUNT3 and HUNT4. If CRP was measured in HUNT4, then it was used in the study, otherwise, CRP from HUNT3 were used. For the rest of measurements, if measured variables from HUNT3 were available, then these were used in the study, otherwise, measurements from HUNT2 were used. Outliers from each study round were excluded from the analyses using the same criteria as in the UKB analyses. BMI and non-fasting glucose, in addition to triglycerides, were natural log-transformed prior to analysis. In HUNT, information on each outcome variable was available in 97.9-99.6 % (insert here the min-max percentages for variables except CRP and HbA1c) of included HUNT participants, except for CRP (84.8%) and HbA1c (64.4%).

**Extraction of blood pressure associated SNPs**

We extracted the imputed dosages for all 68 autosomal independent lead SBP associated SNPs (P < 5x10-8) and 71 DBP associated SNPs from the imputed data provided by the UKB for our analyses (Table S2). All SNPs were well-imputed with information metric (INFO) scores > 0.9 (Table S2). The SNPs were extracted from the UKB imputed genotype data in dosage format using qctool (version 2.0.2, <https://www.well.ox.ac.uk/~gav/qctool/> ). Similarly, all SNPs were available in the imputed genetic data from the HUNT Study, except rs805303 (which is associated with both SBP and DBP), and all were well-imputed (quality metric Rsq > 0.4). The SNPs were extracted from the HUNT imputed genotype data in dosage format using plink2 ^21^.

We used unweighted genetic scores of blood pressure associated SNPs (SBP/DBP) as an instrument for maternal blood pressure during pregnancy, rather than weighted genetic scores or individual SNPs. We used unweighted genetic scores in order to reduce the effect of winner’s curse ^22^, and because weighting according to coefficients derived from a population-based sample may not be appropriate when instrumenting blood pressure during pregnancy.

**Analyses of the association of the selected blood pressure associated SNPs in pregnant women.**

First, we compared the SNP effect sizes for SBP and DBP with those from a GWAS of hypertension during pregnancy in FINNGEN (<http://r2.finngen.fi/> ). Second, we conducted a genetic correlation analysis using linkage disequilibrium (LD)-score regression via the Complex Trait Genetics Virtual Laboratory (CTG-VL) platform ^23^, with summary statistics from the FINNGEN hypertension in pregnancy GWAS and both an SBP and DBP GWAS using automated readings in the UKB from the Neale Lab (<http://www.nealelab.is/uk-Biobank/>). Third, we checked whether an unweighted genetic score of blood pressure associated SNPs was associated with maternal blood pressure during pregnancy (measured at the following gestational ages: 8 weeks, 18 weeks, 30 weeks, and 36 weeks) in the Avon Longitudinal Study of Parents and Children (ALSPAC) ^24^ using linear regression. We also calculated the percentage of variance in maternal blood pressure explained by the unweighted genetic score in ALSPAC.

Our selected blood-pressure associated SNPs showed a relatively consistent direction of effect between the FinnGen GWAS of gestational hypertension and the GWAS of SBP/DBP in non-pregnant population based samples of individuals. Only one of the 94 independent SNPs (excluding overlapping SNPs between SBP-associated and DBP-associated SNPs) had a nominally significant effect in the opposite direction of association for gestational hypertension compared to DBP in the non-pregnant population based samples (Figure S2). Consistent with this, there was a strong genetic correlation between hypertension during pregnancy and both SBP and DBP in participants from the non-pregnant general population, as estimated using LD-score regression ^25,26^ (SBP: rG = 0.71, [95% CI: 0.40- 1.02]; DBP: rG = 0.76, [95% CI: 0.43- 1.09], Table S4).

We found strong evidence that the unweighted maternal genetic scores of the blood pressure variants were positively associated with higher maternal blood pressure during pregnancy in ALSPAC (P < 10-10, Table S5). The unweighted maternal genetic scores explained 0.7% of the variance in both SBP and DBP during pregnancy, which was comparable to the variance explained in blood pressure (SBP/DBP) of the non-pregnant mothers in their middle-to-late adulthood used in the association analysis from UKB and HUNT (0.7% and 0.8%, respectively).

**Regression analyses in UKB and HUNT**

In the UKB study, we directly tested the association in an ordinary least-squares (OLS) linear regression model between maternal genetic score and offspring outcomes in up to 3,756 mother-offspring pairs, adjusting for the offspring’s genetic score calculated form the same blood pressure associated SNPs (Figure S1B). All analyses were adjusted for offspring age at measurement, sex, assessment centre and the top 40 genetic PCs provided by UKB.

In the HUNT study, we conducted similar association analyses in up to 25,952 mother-offspring pairs for cardiometabolic variables that were available in this cohort. Unlike UKB, there is a large amount of relatedness between participants in the HUNT study^4^. This statistical analyses were performed in R (version 3.5.3) ^27^. We have previously described a method that accounts for the genetic relatedness using full information maximum likelihood (FIML), thus avoiding the need to exclude related participants ^4^. In short, we fit a linear mixed model where relatedness was parameterized by a genome-wide genetic relationship matrix to account for the cryptic relatedness across offspring in the sample. Tests for association were performed in the fixed effects part of the model where maternal and offspring genetic scores were fitted as fixed effects. This statistical model was run using the OpenMx package (version 2.13.2) in R (version 3.5.3) ^27^. In addition to conditioning on the offspring’s genetic score and genetic relatedness, all analyses were adjusted for offspring age at measurement, sex, and study round (if measurements from multiple rounds were included).

**Sensitivity analyses in UKB mother-offspring pairs**

To ensure our results were not influenced by violations of the normality of errors assumption, we conducted a sensitivity analysis using an inverse normal transformation for all offspring outcomes. The analysis using inverse normal transformed outcomes did not make an appreciable difference in the direction nor statistical significance of these associations (Table S7).

To compare any differences between models using unweighted and weighted genetic scores for maternal blood pressure (i.e. difference in explained variance of blood pressure), we conducted sensitivity analyses using OLS linear regression of offspring cardiometabolic phenotypes on maternal/paternal weighted genetic score in the UKB, conditional on offspring individual genotypes at the same blood pressure loci. The betas for construction of the weighted genetic score were extracted from external blood pressure GWAS of male and non-pregnant female participants (Table S2).

In the OLS linear regression analysis using maternal and paternal weighted genetic scores for SBP and DBP in the UKB, the same direction of association of similar magnitude was detected for all offspring cardiometabolic traits (Table S10 - S11).

**Sensitivity analyses in HUNT mother-offspring pairs**

The offspring in the HUNT study had a wider age range (20 - 85 years) than UKB (39 - 54 years), with an average age of 40 years at the time of measurement. It is possible that younger participants within the HUNT Study did not show observable changes in cardiometabolic risk factors, reducing the statistical power of our analyses to detect evidence of a causal association between maternal blood pressure and the cardiometabolic risk factors in middle-to-late adulthood. Therefore, we conducted a secondary analysis dividing the data into two strata based on the age of the offspring (offspring < 40 years of age and offspring > 40 years of age), with the older age strata being similar to the age distribution in the UKB.

Evangelou et al. ^28^ published the largest GWAS of blood pressure to date including over 1 million participants. We did not use this GWAS for instrument selection in the UKB analysis because it included participants from UKB in the discovery stage; however, it did not include the HUNT study. We, therefore, conducted additional analyses in the HUNT Study using unweighted genetic scores comprised of 244 SBP-associated SNPs and 323 DBP-associated SNPs identified by Evangelou et al. (Table S3). The proportion of variance in blood pressure explained by the unweighted genetic scores was also calculated in ALSPAC mothers and HUNT mothers.

Given the majority of the UKB participants were older than 40 years of age, we conducted an age stratified analysis in the HUNT study to determine whether the association was driven by older participants who were more likely to have developed an adverse cardiometabolic profile. However, no significant associations were observed in the age stratified analyses in mother-offspring pairs (Table S12) or father-offspring pairs (Table S13).

Using the larger set of SNPs extracted from the GWAS of Evangelou et al., the unweighted genetic score explained 0.5% of SBP during pregnancy in the ALSPAC mothers and 1.0% in DBP. This is less than the variance explained in blood pressure of the non-pregnant mothers in their middle-to-late adulthood from HUNT (1.3% and 1.4% for SBP and DBP, respectively). A higher maternal SBP genetic score, but not DBP genetic score, was nominally associated with higher levels of offspring’s non-fasting glucose (log-transformed, beta= 0.0003, [95% CI: 0 - 0.0005], P = 0.0271) and higher TG (log-transformed, beta= 0.0008, [95% CI: 0 - 0.0015], P = 0.0496) using this larger set of SNPs (Table S14). A similar pattern of association was also observed in > 40 years strata, but not in < 40 years strata (Table S13). The genetic scores were not associated with non-fasting glucose or TG in father-offspring pairs (Table S14). However, these nominal associations were not observed in the primary meta-analysis or individual cohort analysis of HUNT and UKB which used a smaller set of genetic variants (Table 1). Neither directions nor magnitudes of effect estimates were consistent across all analyses. This indicates that the suggestive associations from analyses using more genetic variants might represent false positives (type 1 errors).

**Sensitivity analyses father-offspring pairs**

Associations between maternal blood pressure associated genetic variants and offspring outcomes may reflect effects mediated through the intrauterine or postnatal environment. If the effect is entirely mediated through the intrauterine environment, then we might expect to see an association between maternal genetic variants and offspring phenotypes, but not between paternal genetic variants at the same loci and offspring outcomes. In contrast, if the effect is mediated through the postnatal environment, then we might expect to see associations between both maternal and paternal genetic variants and offspring phenotypes (with the caveat that paternal programming via changes to sperm epigenetics or seminal fluid provides an alternative explanation for associations between paternal genetic variants and offspring phenotypes ^29^). In order to investigate these possibilities, we also conducted similar OLS linear regression analyses in up to 1,703 father-offspring pairs from the UKB and 19,720 father-offspring pairs from the HUNT Study. A heterogeneity test of the beta coefficients estimated in the analyses for the maternal and paternal genetic scores was conducted to compare the direction and magnitude of the effect. The heterogeneity test was conducted using Rmeta package (version 3.0) in R (version 3.5.3).

**Power calculation**

We assumed N = 29,708 mother-offspring pairs in total (N=3,756 and N = 25,952 from UKB and HUNT, respectively), the absence of maternal phenotypes, and a two sided Type 1 error rate of α = 0.05. Figure S3 illustrates the relationship between maternal genetics and offspring genetics, the maternal blood pressure (modelled as a single latent unobserved variable), and offspring cardiometabolic traits. In this diagram, we assume that no effect of maternal genetics on the offspring cardiometabolic risk factor goes through paths other than maternal blood pressure (i.e. no pleiotropy). To make calculations and explication easier, we assume that all variables have been standardized to unit variance. Under this model, the pathway from maternal genetics to offspring cardiometabolic traits via offspring genetics was blocked in the analysis. So only two elements contribute to the variance of offspring cardiometabolic traits. One is the variance explained by maternal genetics associated with blood pressure (i.e. γ^2^λ^2^). The second is the residual variance of cardiometabolic risk factors (variance of ε). This latter term includes both environmental and genetic factors that are not modelled in the study. The variance in cardiometabolic risk factors explained by the maternal genetics is a function of the direct association between the SNPs and the maternal blood pressure (the path coefficient γ), and the effect of the maternal blood pressure on cardiometabolic risk factor (the path coefficient λ- the precise formula being: γ^2^λ^2^). There is no reference for phenotypic correlations between maternal blood pressure during pregnancy and offspring cardiometabolic risk factors since the blood pressure during pregnancy is absent in both cohorts. We assume the proportion of variance explained in maternal blood pressure by maternal genetics is 0.8% (γ^2^). To give the reader an idea of the potentially small numbers involved, we assume the path coefficient the λ =$\sqrt{0.02125}$ = 0.1458. These values, in turn, would imply that the variance explained in the cardiometabolic risk factor by the maternal genetic risk score would be 0.008 x 0.02125 = 0.017%, which is a small proportion of the variance, and one that we are moderate powered to detect (>50%) in the current study (N = 29708, Table S15).

References

1. Fry A, Littlejohns TJ, Sudlow C, Doherty N, Adamska L, Sprosen T, Collins R, Allen NE. Comparison of Sociodemographic and Health-Related Characteristics of UK Biobank Participants With Those of the General Population. *American journal of epidemiology*. 2017;186:1026-1034. doi: 10.1093/aje/kwx246

2. Bycroft C, Freeman C, Petkova D, Band G, Elliott LT, Sharp K, Motyer A, Vukcevic D, Delaneau O, O'Connell J, et al. The UK Biobank resource with deep phenotyping and genomic data. *Nature*. 2018;562:203-209. doi: 10.1038/s41586-018-0579-z

3. Ferreira MA, Vonk JM, Baurecht H, Marenholz I, Tian C, Hoffman JD, Helmer Q, Tillander A, Ullemar V, van Dongen J, et al. Shared genetic origin of asthma, hay fever and eczema elucidates allergic disease biology. *Nat Genet*. 2017;49:1752-1757. doi: 10.1038/ng.3985

4. Moen GH, Brumpton B, Willer C, Asvold BO, Birkeland KI, Wang G, Neale MC, Freathy RM, Smith GD, Lawlor DA, et al. Mendelian randomization study of maternal influences on birthweight and future cardiometabolic risk in the HUNT cohort. *Nat Commun*. 2020;11:5404. doi: 10.1038/s41467-020-19257-z

5. Boyd A, Golding J, Macleod J, Lawlor DA, Fraser A, Henderson J, Molloy L, Ness A, Ring S, Davey Smith G. Cohort profile: the ‘children of the 90s’—the index offspring of the Avon Longitudinal Study of Parents and Children. *Int J Epidemiol*. 2013;42:111-127. doi:

6. Fraser A, Macdonald-Wallis C, Tilling K, Boyd A, Golding J, Davey Smith G, Henderson J, Macleod J, Molloy L, Ness A. Cohort profile: the Avon Longitudinal Study of Parents and Children: ALSPAC mothers cohort. *Int J Epidemiol*. 2013;42:97-110. doi:

7. Harris PA, Taylor R, Thielke R, Payne J, Gonzalez N, Conde JG. Research electronic data capture (REDCap)—a metadata-driven methodology and workflow process for providing translational research informatics support. *Journal of biomedical informatics*. 2009;42:377-381. doi:

8. 1000 Genomes Project Consortium, Auton A, Brooks LD, Durbin RM, Garrison EP, Kang HM, Korbel JO, Marchini JL, McCarthy S, McVean GA, et al. A global reference for human genetic variation. *Nature*. 2015;526:68-74. doi: 10.1038/nature15393

9. Abraham G, Qiu Y, Inouye M. FlashPCA2: principal component analysis of Biobank-scale genotype datasets. *Bioinformatics*. 2017;33:2776-2778. doi: 10.1093/bioinformatics/btx299

10. Wang C, Zhan X, Bragg-Gresham J, Kang HM, Stambolian D, Chew EY, Branham KE, Heckenlively J, Study F, Fulton R, et al. Ancestry estimation and control of population stratification for sequence-based association studies. *Nat Genet*. 2014;46:409-415. doi: 10.1038/ng.2924

11. Li JZ, Absher DM, Tang H, Southwick AM, Casto AM, Ramachandran S, Cann HM, Barsh GS, Feldman M, Cavalli-Sforza LL, et al. Worldwide human relationships inferred from genome-wide patterns of variation. *Science*. 2008;319:1100-1104. doi: 10.1126/science.1153717

12. Manichaikul A, Mychaleckyj JC, Rich SS, Daly K, Sale M, Chen WM. Robust relationship inference in genome-wide association studies. *Bioinformatics*. 2010;26:2867-2873. doi: 10.1093/bioinformatics/btq559

13. International Consortium for Blood Pressure Genome-Wide Association Studies, Ehret GB, Munroe PB, Rice KM, Bochud M, Johnson AD, Chasman DI, Smith AV, Tobin MD, Verwoert GC, et al. Genetic variants in novel pathways influence blood pressure and cardiovascular disease risk. *Nature*. 2011;478:103-109. doi: 10.1038/nature10405

14. Tobin MD, Sheehan NA, Scurrah KJ, Burton PR. Adjusting for treatment effects in studies of quantitative traits: antihypertensive therapy and systolic blood pressure. *Stat Med*. 2005;24:2911-2935. doi: 10.1002/sim.2165

15. Boekholdt SM, Arsenault BJ, Mora S, Pedersen TR, LaRosa JC, Nestel PJ, Simes RJ, Durrington P, Hitman GA, Welch KM, et al. Association of LDL cholesterol, non-HDL cholesterol, and apolipoprotein B levels with risk of cardiovascular events among patients treated with statins: a meta-analysis. *Jama*. 2012;307:1302-1309. doi: 10.1001/jama.2012.366

16. Locke AE, Steinberg KM, Chiang CWK, Service SK, Havulinna AS, Stell L, Pirinen M, Abel HJ, Chiang CC, Fulton RS, et al. Exome sequencing of Finnish isolates enhances rare-variant association power. *Nature*. 2019;572:323-328. doi: 10.1038/s41586-019-1457-z

17. Liu DJ, Peloso GM, Yu H, Butterworth AS, Wang X, Mahajan A, Saleheen D, Emdin C, Alam D, Alves AC, et al. Exome-wide association study of plasma lipids in >300,000 individuals. *Nat Genet*. 2017;49:1758-1766. doi: 10.1038/ng.3977

18. Asselbergs FW, Guo Y, van Iperen EP, Sivapalaratnam S, Tragante V, Lanktree MB, Lange LA, Almoguera B, Appelman YE, Barnard J, et al. Large-scale gene-centric meta-analysis across 32 studies identifies multiple lipid loci. *American journal of human genetics*. 2012;91:823-838. doi: 10.1016/j.ajhg.2012.08.032

19. Horikoshi M, Beaumont RN, Day FR, Warrington NM, Kooijman MN, Fernandez-Tajes J, Feenstra B, van Zuydam NR, Gaulton KJ, Grarup N, et al. Genome-wide associations for birth weight and correlations with adult disease. *Nature*. 2016;538:248-252. doi: 10.1038/nature19806

20. Friedewald WT, Levy RI, Fredrickson DS. Estimation of the concentration of low-density lipoprotein cholesterol in plasma, without use of the preparative ultracentrifuge. *Clin Chem*. 1972;18:499-502. doi:

21. Chang CC, Chow CC, Tellier LC, Vattikuti S, Purcell SM, Lee JJ. Second-generation PLINK: rising to the challenge of larger and richer datasets. *Gigascience*. 2015;4:7. doi: 10.1186/s13742-015-0047-8

22. Kraft P. Curses--winner's and otherwise--in genetic epidemiology. *Epidemiology*. 2008;19:649-651; discussion 657-648. doi: 10.1097/EDE.0b013e318181b865

23. Cuellar-Partida G, Lundberg M, Kho PF, D’Urso S, Gutierrez-Mondragon LF, Hwang L-D. Complex-Traits Genetics Virtual Lab: A community-driven web platform for post-GWAS analyses. 2019:518027. doi: 10.1101/518027 %J bioRxiv

24. Fraser A, Macdonald-Wallis C, Tilling K, Boyd A, Golding J, Davey Smith G, Henderson J, Macleod J, Molloy L, Ness A, et al. Cohort Profile: the Avon Longitudinal Study of Parents and Children: ALSPAC mothers cohort. *Int J Epidemiol*. 2013;42:97-110. doi: 10.1093/ije/dys066

25. Bulik-Sullivan B, Finucane HK, Anttila V, Gusev A, Day FR, Loh PR, ReproGen C, Psychiatric Genomics C, Genetic Consortium for Anorexia Nervosa of the Wellcome Trust Case Control C, Duncan L, et al. An atlas of genetic correlations across human diseases and traits. *Nat Genet*. 2015;47:1236-1241. doi: 10.1038/ng.3406

26. Bulik-Sullivan BK, Loh PR, Finucane HK, Ripke S, Yang J, Schizophrenia Working Group of the Psychiatric Genomics C, Patterson N, Daly MJ, Price AL, Neale BM. LD Score regression distinguishes confounding from polygenicity in genome-wide association studies. *Nat Genet*. 2015;47:291-295. doi: 10.1038/ng.3211

27. Boker S, Neale M, Maes H, Wilde M, Spiegel M, Brick T, Spies J, Estabrook R, Kenny S, Bates T, et al. OpenMx: An Open Source Extended Structural Equation Modeling Framework. *Psychometrika*. 2011;76:306-317. doi: 10.1007/s11336-010-9200-6

28. Evangelou E, Warren HR, Mosen-Ansorena D, Mifsud B, Pazoki R, Gao H, Ntritsos G, Dimou N, Cabrera CP, Karaman I, et al. Genetic analysis of over 1 million people identifies 535 new loci associated with blood pressure traits. *Nat Genet*. 2018;50:1412-1425. doi: 10.1038/s41588-018-0205-x

29. Lane M, Robker RL, Robertson SA. Parenting from before conception. *Science*. 2014;345:756-760. doi: 10.1126/science.1254400

30. Evans DM, Moen GH, Hwang LD, Lawlor DA, Warrington NM. Elucidating the role of maternal environmental exposures on offspring health and disease using two-sample Mendelian randomization. *Int J Epidemiol*. 2019;48:861-875. doi: 10.1093/ije/dyz019

**Supplementary Tables**

**Table S1-S15**

Please find the tables in the separate file.

**Supplementary Figures**


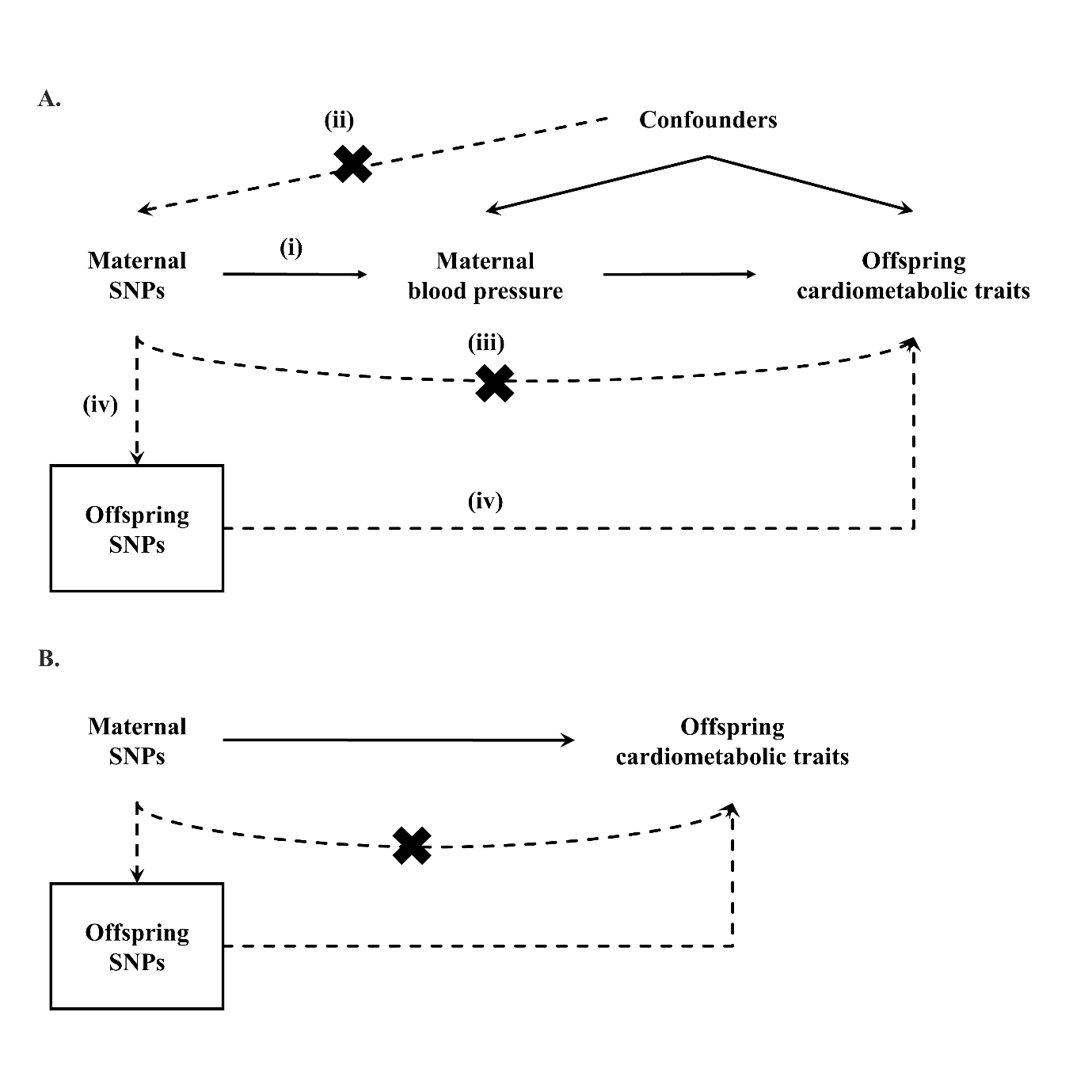


**Figure S1** A. Directed acyclic graph illustrating the core assumptions underlying Mendelian randomization studies of maternal exposures and offspring outcomes. Assumption (i) requires a reliable association between the genetic variants and the maternal exposure (blood pressure). Assumption (ii) requires that the genetic variants must not be associated with confounders of the maternal exposure-offspring outcome relationship. Assumption (iii) assumes no pathway from maternal genetic variants to offspring outcomes that do not pass through the maternal exposure of interest. Genetic variants inherited by the offspring from their mother violate assumption (iii) if they also affect the offspring phenotype (iv). However, conditioning on offspring variants (indicated by the box around offspring SNPs) blocks the pathway (iv) (indicated by the cross on the arrow). (Adapted from Evans et al., Int J Epidemiol, 2019^30^). It is assumed that paternal variants at the same locus do not affect the offspring outcome and consequently that conditioning on offspring genotype will not induce an open collider path between maternal genotype, paternal genotype and offspring outcome; B. Directed acyclic graph illustrating the ordinary least squares linear regression analysis in the current study. Paths involving genetic transmission from mother to offspring genome were controlled by conditioning on offspring variants at the same loci (indicated by the box around offspring SNPs).

**Figure S2** Genetic association study results for 94 independent blood pressure associated SNPs (excluding overlapping SNPs between SBP-associated and DBP-associated SNPs) in pregnant women from the FINNGEN GWAS of gestational hypertension*. The y axis shows the allelic effect sizes (beta coefficients) for gestational hypertension (with standard error bars) per blood pressure increasing allele. The x axis displays the name of the SNP markers in ascending order of P value from the FINNGEN GWAS of hypertension. 25 SNPs have nominally significant effects in the same direction as the SBP/DBP GWAS (marked in black, lower bound of 95% confidence interval > 0). Only one SNP had a nominally significant effect in the opposite direction (marked in red). 68 SNPs have 95% confidence intervals that span zero. Five SNPs were not reported in the FINNGEN study (rs11229457, rs1975487, rs2187668, rs2898290, rs687621).

*Hypertension complicating pregnancy, childbirth, and the puerperium: Includes ICD-10 codes: O10: Pre-existing hypertension complicating pregnancy, childbirth and the puerperium, O11: Pre-existing hypertensive disorder with superimposed proteinuria, O13: Gestational [pregnancy-induced] hypertension without significant proteinuria, O14: Gestational [pregnancy-induced] hypertension with significant proteinuria, O15: Eclampsia, O16: Unspecified maternal hypertension. FINNGEN: 3362 cases, 50900 controls (<http://r2.finngen.fi/pheno/I9_HYPTENSPREG>)


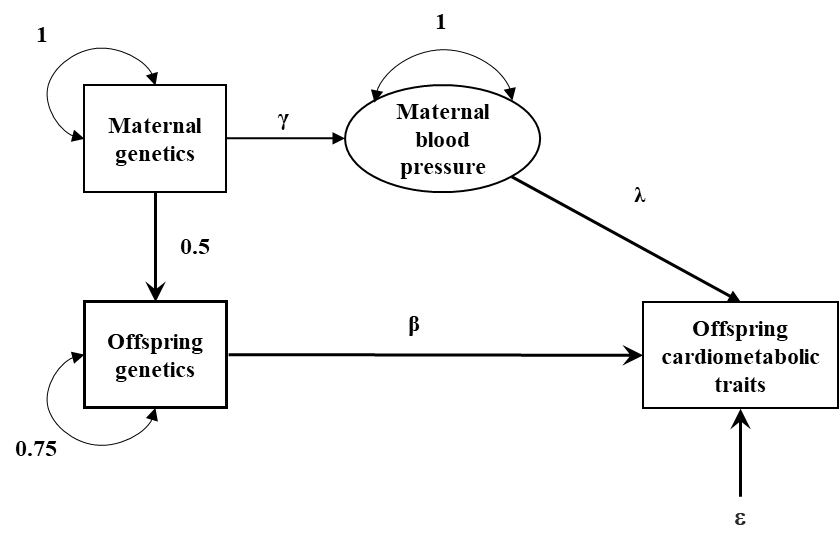


**Figure S3** Path diagram of the relationship between maternal genetics and offspring genetics, maternal blood pressure and offspring cardiometabolic traits. Variables within square boxes represent observed variables, whereas variables in circles represent latent unobserved variables. Unidirectional arrows represent causal relationships from tail to head, whilst two-headed arrows represent correlational relationships. Greek letters or numbers on one headed arrows represent path coefficients which quantify the expected causal effect of one variable on the other. Numbers on two-headed arrows represent covariances between variables. The epsilon variable represents residual latent factors (both environmental and genetic) that are not modelled in the study. We assume that all variables are standardized to have unit variance. Consequently, the residual variance of the offspring genetics is set to 0.75 since ¼ of the variance comes from the maternal genotype. The pathway from maternal genetics to offspring cardiometabolic traits via offspring genetics was blocked in the MR analysis. For the purposes of the power calculation described in the discussion, we assume that maternal single nucleotide polymorphisms exert long term effects on the offspring cardiometabolic risk factor of interest.


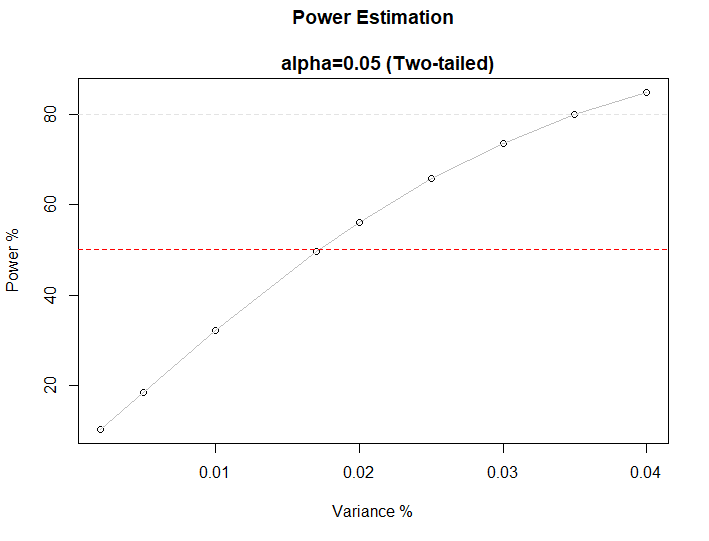


**Figure S4** Statical power to detect maternal genetic effects. The y axis displays statistical power to detect association (α = 0.05, two tailed test) as estimated by the Maternal and Offspring Genetic Effects Power Calculator (<https://evansgroup.di.uq.edu.au/MGPC/>) using the number of genotyped mother-offspring pairs in the combined UKB and HUNT cohorts (N = 29813). The x axis displays the proportion of variance explained (%) in the offspring phenotype by maternal genetic effects. Each point on the graph displays the results of one power calculation with parameters corresponding to Table S15. The light grey and red dashed lines indicate 80% and 50% power to detect maternal genetic effects respectively.
